# Smartphone Biosensor System with Multi-Testing Unit Based on Localized Surface Plasmon Resonance Integrated with Microfluidics Chip

**DOI:** 10.3390/s20020446

**Published:** 2020-01-13

**Authors:** Zhiyuan Fan, Zhaoxin Geng, Weihao Fang, Xiaoqing Lv, Yue Su, Shicai Wang, Hongda Chen

**Affiliations:** 1 State Key Laboratory of Integrated Optoelectronics, Institute of Semiconductors, Chinese Academy of Sciences, Beijing 100083, China; Fanzhiyuan@semi.ac.cn (Z.F.); fwh@semi.ac.cn (W.F.); lvxiaoqing284@semi.ac.cn (X.L.); suyue@semi.ac.cn (Y.S.); hdchen@semi.ac.cn (H.C.); 2 Center of Materials Science and Optoelectronics Engineering, University of Chinese Academy of Sciences, Beijing 100049, China; 3 School of Information Engineering, Minzu University of China, Beijing 100081, China; 4 State Key Laboratory of Crystal Materials, Shandong University, Jinan 250100, China; cjchao@semi.ac.cn

**Keywords:** localized surface plasmon resonance, smartphone, multipoint biosensor, microfluidics, point-of-care, protein detection

## Abstract

Detecting biomarkers is an efficient method to diagnose and monitor patients’ stages. For more accurate diagnoses, continuously detecting and monitoring multiple biomarkers are needed. To achieve point-of-care testing (POCT) of multiple biomarkers, a smartphone biosensor system with the multi-testing-unit (SBSM) based on localized surface plasmon resonance (LSPR) integrated multi-channel microfluidics was presented. The SBSM could simultaneously record nine sensor units to achieve the detection of multiple biomarkers. Additional 72 sensor units were fabricated for further verification. Well-designed modularized attachments consist of a light source, lenses, a grating, a case, and a smartphone shell. The attachments can be well assembled and attached to a smartphone. The sensitivity of the SBSM was 161.0 nm/RIU, and the limit of detection (LoD) reached 4.2 U/mL for CA125 and 0.87 U/mL for CA15-3 through several clinical serum specimens testing on the SBSM. The testing results indicated that the SBSM was a useful tool for detecting multi-biomarkers. Comparing with the enzyme-linked immunosorbent assays (ELISA) results, the results from the SBSM were correlated and reliable. Meanwhile, the SBSM was convenient to operate without much professional skill. Therefore, the SBSM could become useful equipment for point-of-care testing due to its small size, multi-testing unit, usability, and customizable design.

## 1. Introduction

Point-of-care testing (POCT) is booming in recent years due to the great need for fast, easy, portable, and precise biosensor technology. POCT allows doctors to get precise results in clinics, homes, and even fields rather than hospitals or laboratories. POCT also is designed for patients who have not been specifically trained to get self-test at home. The POCT equipment should be portable so that doctors and patients could use it anywhere [1,2,3,4,5]. The advantages of POCT could help doctors to continuously monitor the health state of patients, which is necessary for some chronic diseases such as cancers, diabetes, and heart diseases. As essential equipment for people nowadays, the smartphone is regarded as one of the most suitable platforms for POCT. Firstly, smartphones usually are pocket-sized, which meets the need of portability for POCT. Secondly, smartphones have integrated multi-sensors such as optical sensors, magnetic field sensors, gyroscopes, and so on. These sensors can be applied in POCT with some kits and chips [6,7,8,9]. Last but not least, the smartphone is a communication tool. The ability to keep online allows smartphone users to upload their health data immediately. Depending on the technology of cloud computing and big data, these health data can be analyzed fast and precisely. Therefore, doctors could receive results and give out suggestions without meeting patients. As a booming research field, many methods of biosensing have been achieved on the smartphone. However, the complementary metal-oxide-semiconductor (CMOS) image sensors are the most applicated sensors in the smartphone-based POCT. Owing to the powerful ability to record the light signal, the CMOS image sensors on smartphones could be alternative equipment for optical biosensing.

Optical biosensing, including enzyme-linked immunosorbent assays (ELISA), fluorescence, surface plasmon resonance (SPR), localized surface plasmon resonance (LSPR), have been widely used in laboratories and hospitals to detected biomarkers [10,11]. Conventional optical biosensor systems in laboratories and hospitals usually have a bulk system, several fiber connections, an expensive spectrometer, and a computer as a spectral analyzer. These devices are usually hard to assemble and use without professional training. In past decades, tremendous effort has been devoted by researchers to achieve optical POCT on smartphone platforms [8,9]. Smartphones could act as a microscope with the help of a lens system. The smartphone-based microscope may be the earliest smartphone-based biosensor [12,13]. With the development of cameras on the smartphone, pictures taken by smartphones become more and more distinguishable. The fluorescence-based and colorimetric-based biosensing are applied on smartphones. The color information of photos taken by smartphones can be analyzed immediately using a designated smartphone application (APP). Next, the result of biosensing could be presented on the screen of the smartphone, clearly and directly [14,15,16,17,18]. For more precise information, the spectrum of the light signal should be made out. Therefore, spectroscopy-based biosensors were widely used in laboratories and hospitals due to their high sensitivity and low limit of detection (LoD). With the help of a grating, the spectral information of light could be captured by CMOS image sensors on smartphones. The spectroscopy-based biosensing, such as ELISA, SPR, LSPR, have been applied on the smartphone [19,20,21,22,23,24]. ELISA, which is usually used in laboratories, is complicated for untrained people. SPR, which has the highest sensitivity, needs a complex measurement system. LSPR, which is highly sensitive as well as user-friendly, maybe the best choice for spectroscopy-based biosensing on the smartphone platform.

Lots of attempts have been made to apply LSPR based biosensor on the smartphone platform. Dutta at al. presented an LSPR-based biosensor on the smartphone platform [22]. However, they used cuvette to measure the spectrum of the solution, which needed a high volume of samples. Wang at al. reported an LSPR chip-based biosensor on the smartphone platform [25]. The nanohole chip needed fewer samples than cuvette, but the attachments used in Wang’s work was big, which did not meet the needs of POCT. Wang at al. presented the first multichannel smartphone spectrometer as an optical biosensor [24]. However, the multichannel smartphone spectrometer was applied to ELISA testing. The multichannel smartphone spectrometer was designed to 96-well plates, which was not portable enough.

Here, we demonstrate a smartphone biosensor system with the multi-testing-unit (SBSM) based on localized surface plasmon resonance (LSPR) integrated multichannel microfluidics. The SBSM consisted of a smartphone and some well-designed modularized attachments. The attachments have a smaller volume than other reported multichannel smartphone spectrometers, and the attachments could be replaced easily to suit any model of smartphones. The sensor chip applied in the SBSM is based on LSPR and microfluidics. Multichannel spectrometer biosensor based on LSPR was firstly applied on smartphone platforms. For each microchannel, the usage of the sample was only 10 μL, which is much less than 96-well plates (about 300 μL). Each chip has nine microfluidics channels and 81 sensor unit for the detection of multiple biomarkers. Finally, a program was written to read and analyze the spectral information.

## 2. Materials and Methods

### 2.1. Materials

Quartz substrate (JGS1) was purchased from Wuxi Crystal and Optical Instrument Co. Ltd., China. Sulfuric acid, hydrogen peroxide, and sucrose were purchased from Sinopharm Chemical Reagent Beijing, Co. Ltd., China. Deionized (DI) water was purchased from Solarbio Beijing, Co. Ltd., China. Ethanol was purchased from Shanghai Aladdin Bio-Chem Technology Co., Ltd., China. Polydimethylsiloxane (PDMS) was obtained from Dow Corning, USA. Ethanolamine, thiolated alkane 10-carboxy-1-decanethiol (11-MUA), 1-ethyl-3-(3-dimethylamino-propyl) carbodiimide-HCl (EDC), N-hydroxy succinimide (NHS), and bovine serum albumin (BSA) were purchased from Sigma–Aldrich, USA. Phosphate buffer solution (PBS: 0.0067 M, pH: 7.4) was purchased from Thermo Fisher, USA. Cancer antigen 125 protein (CA125, MUC16-1H) and cancer antigen 15-3 (CA15-3, MUC1-376H) were purchased from Creative BioMart, USA. Anti-MUC16 antibody (for CA125, ab134093) and anti-MUC1 antibody (for CA15-3, ab109185) were purchased from Abcam, USA. More information about materials, chemicals, and biologicals was listed in Table S1.

### 2.2. Substrate Preparation and Gold Nanoparticle Fabrication

Quartz chip acted as the substrate, whose size is 20 × 20 × 0.5 mm. To obtain a clean and dry surface for gold nanoparticles (AuNPs) fabrication, the substrate should be pretreated. The substrate was immersed in the piranha solution at 180 °C for 30 min and rinsed by deionized water for about 30 times to remove organic contaminations. Nitrogen gas and hotplate were successively used to remove residual water on the substrate.

**Figure 1:**
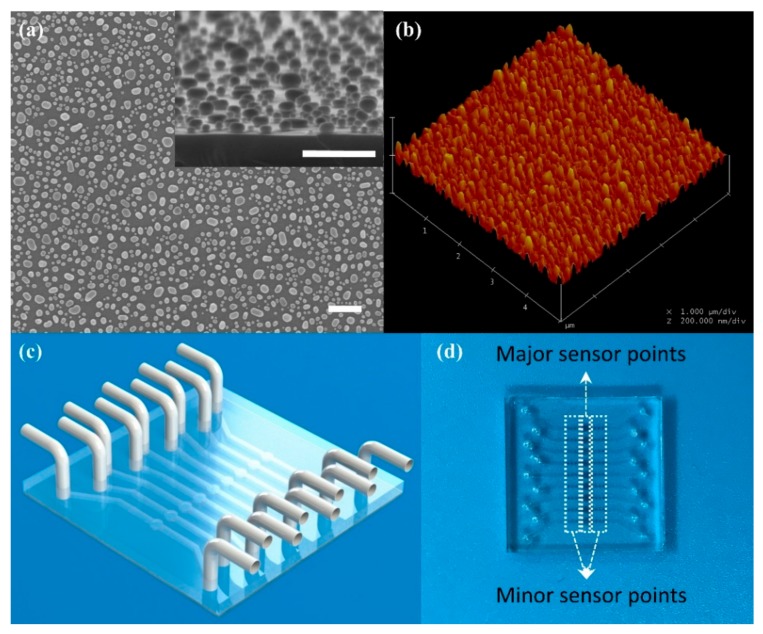
Schematic of the chip integrated with the microfluidic channel and picture of AuNPs. (**a**) The SEM (scanning electron microscope) image of AuNPs on a silicon substrate. Insert Figure: side view of AuNPs on a silicon substrate. Scale bar: 300 nm; (**b**) The AFM (atomic force microscope) image of AuNPs; (**c**) Schematic of the microfluidic chip; (**d**) The real picture of the chip.

The AuNPs were fabricated via annealing method. Gold film (8 nm thick) was deposited on the substrate via electron beam evaporation. A metal mask was used to define a sensing array. The 9 × 9 array was divided into nine groups by column. Each group had nine sensor units, including a major sensor unit in the middle and eight minor sensor units, distributed on both sides. All sensor units with the thick gold film were annealed at 560 °C for 5 h. After annealing, the gold film on the substrate turned into dispersive nanoparticles, which had properties of LSPR. The AuNPs were measured by SEM (scanning electron microscope) and AFM (atomic force microscope), and the results were presented in Figure 1a,b. The SEM results obtained from a silicon substrate because the quartz substrate is non-conducting material. AuNPs on the silicon substrate were formed through the same processing as AuNPs on the quartz substrate. However, there was a broad variation on the size of AuNPs presented in Figure 1a. Although the uniform AuNPs fabricated via other methods may increase the sensitivity of the LSPR-based sensors [26], the anneal method was a cheaper way to obtain an LSPR-based chip. 

Finally, a designed PDMS cover with micro-channel was boning with the substrate to form the biosensor chip. The PDMS microfluidic cover was replicated by a silicon mold, which was designed to match the metal mask. Nine groups of sensor units were covered by nine micro-channels, respectively, and nine circular cavities on the PDMS microfluidic cover were aligned to nine major sensor units, respectively. The schematic and the photo of the chip were presented in Figure 1c,d. For quantity production, the microfluidic chip could be fabricated via polymer injection molding [27,28], which is a low-cost and fast method. 

### 2.3. Attachments of the SBSM

**Figure 2:**
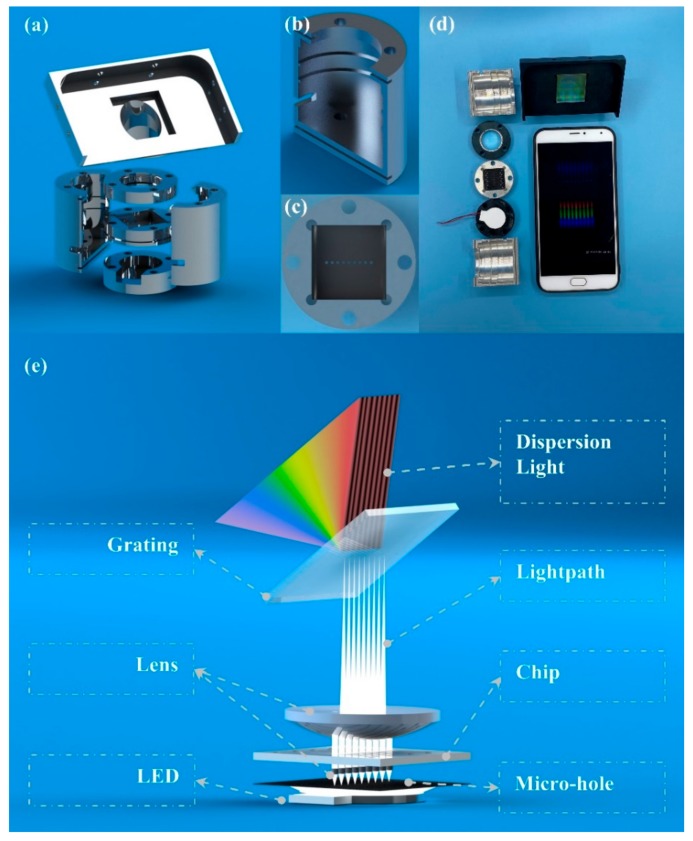
Schematic of attachments of the SBSM (smartphone biosensor system with the multi-testing-unit). (**a**) The schematic of attachments; (**b**) The detail of the case; (**c**) The detail (top view) of the stage of micro-hole array and small-lens array; (**d**) The picture of attachments and the smartphone; (**e**) The working principle of the SBSM.

The attachment of the SBSM was a well-designed measurement system, which consisted of a case, a smartphone stage, three cylindrical stages, and a smartphone shell. The 3D models of all attachments were presented in Figure 2a. The case was a hollow cylinder with several grooves and holes, which was presented in Figure 2b. The position and size of these grooves were suitable for three cylindrical stages, and the holes were used to align all stages. 

Components used in the SBSM included an electric source, a LED light source, nine small lenses (1.0 mm Dia. × 2.0 mm FL, VIS-EXT Coated, Edmund Optics Inc., Barrington, USA), a micro-hole array, a large lens (20.0 mm Dia. × 25.0 mm FL, VIS-EXT Coated, Edmund Optics Inc., Barrington, USA), and a transmission grating (1200 Grooves/mm, Edmund Optics Inc., Barrington, USA). More information about components was listed in Table S1.

The electric source was two alkaline batteries, which could provide 3 V voltage to the LED light source via wires. 

The LED light source was placed in the first cylindrical stage. The light source could directly affect the distribution of light. The LED array was not suitable for the SBSM due to its non-uniform light distribution, which may bring bright spots on photos. Therefore, a backscattering LED light source was applied to overcome the problem of bright spots [24]. 

The second stage had an array of cylindrical grooves. Nine small lenses were placed in grooves in the second stage, which was presented in Figure 2c. The micro-hole array was made of printed film, which was stuck on the second stage. Additionally, the second stage has a square groove to place the biosensor chip. All arrays, including the array of lenses, the array of micro-holes, and the array of major sensor units on the chip were aligned. The array of micro-holes and the array of small lenses could separate the light into nine parts. Therefore, each major sensor unit was illuminated by an independent light path.

The third stage was a hollow cylinder, where the lager lens was placed. The large lens can make converge the light paths on the smartphone camera. Therefore, the light through all major sensor units could be caught in a short light path. 

The smartphone stage was placed on the top of the case. The transmission grating placed on a square groove on the smartphone stage. A 36.9° angle was designed between the incident light and the normal direction of the grating. The smartphone was also placed on the stage with the smartphone shell. The transmission grating was right in front of the smartphone camera.

The photos of the SBSM were presented in Figure 2d. The schematic of the light path was presented in Figure 2e. All attachments were easy to assemble and align with the help of the mounting holes. A screwdriver was the only tool needed during assembly. All attachments were modularized; customers could choose different kinds of attachments to achieve different functions. For example, the grating could change to 2400 grooves/mm to achieve higher resolution, and the smartphone shell could be replaced by whichever matched the customer’s smartphone. 

### 2.4. Spectral Calibration

**Figure 3:**
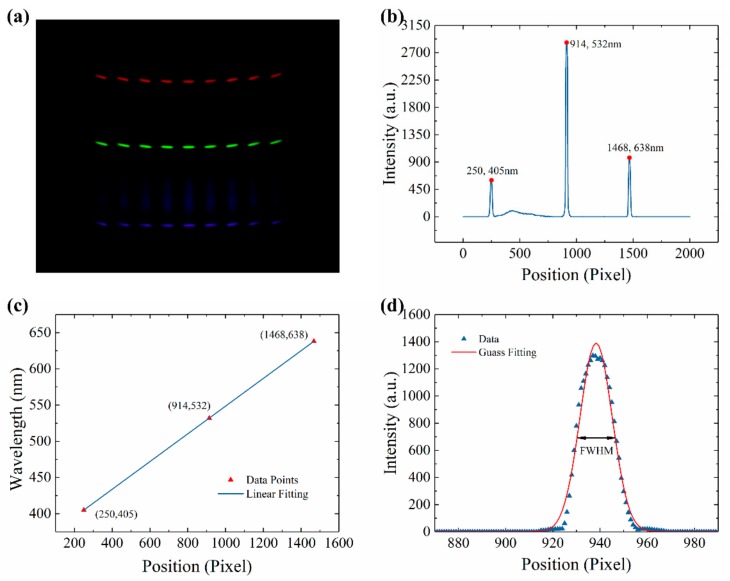
Spectral calibration results. (**a**) Picture of lasers taken by the smartphone; (**b**) Relationship between intensity and position in the picture of lasers; (**c**) Relationship between wavelength and position; (**d**) The spectrum of 532 nm laser and the Gauss fitting curve.

Since the principle of LSPR-based chip was to detect wavelength shift, the spectral calibration was significant. Three lasers, whose center wavelength was 405, 532, and 638 nm, respectively, were used for spectral calibration. Three lasers coupled into the backlight panel through fiber. The picture taken by the smartphone was presented in Figure 3a. Three significant light lines appeared in the picture. A Python program was written to analyze each picture. The workflow of the Python program was presented in Figure S1. The analyzed data was presented in Figure 3b. Based on the center wavelength of lasers and the position of peaks in Figure 3b, the relationship between wavelength and position could be calculated through liner fitting. The fitting result was presented in Figure 3c. The relationship between wavelength and position was as Function (1):

λ = 0.1913x + 357.1695,(1)

where λ represented the wavelength, and x represented the position. The slope value is 0.1913 nm/pixel, which indicated that the index value of the SBSM is 0.1913 nm. The coefficient of determination (R^2^) is nearly equal to 1, which means the accuracy of the SBSM is reliable. 

Due to the limits of the camera on the smartphone, the position of lasers in different channels did not strictly overlap, as shown in Figure 3a. The fitting results of each channel were presented in Table S1. The resolution of the SBSM could be calculated via measuring the full width at half maximum (FWHM) of the 532 nm laser. In Figure 3d, the spectrum of 532 nm laser fitted by Gauss Function. The FWHM of the 532 nm laser was 16.1414 pixels. Since the slope value in Figure 3c was 0.1917 nm/pixel. The FWHM of the 532 nm laser was 3.0878 nm measured by the SBSM. As a reference, the FWHM of the same laser measured by the spectrometer was 1.4 nm. According to the manual of the spectrometer (QE65000, Ocean Optics, Florida, US), the real resolution is 0.32 nm (1200 grating and 10 μm slit). Therefore, it can be estimated that the resolution of the SBSM is 0.71 nm. However, the resolution of the SBSM could improve by applying smaller holes or better grating. However, it will add cost to get higher resolution. Moreover, higher resolution always means lower sensitivity to light.

### 2.5. Analyzation of the Data

**Figure 4:**
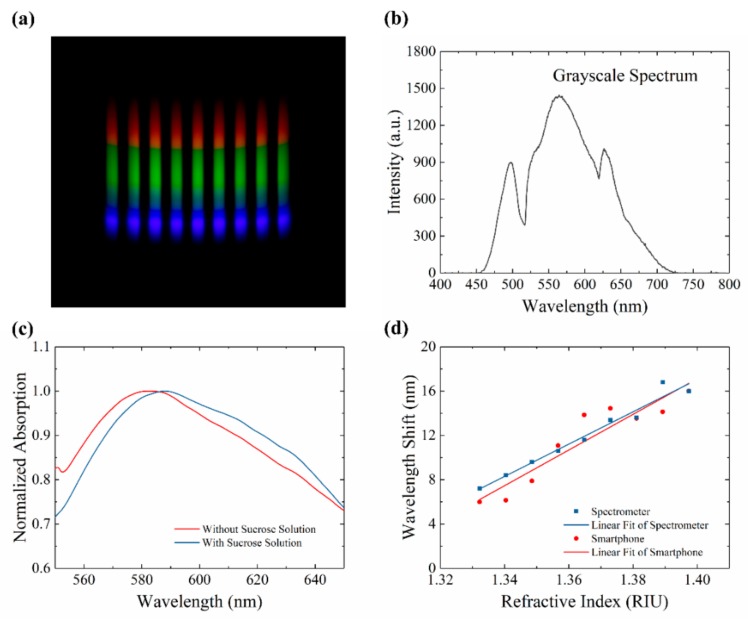
Results of refractive index measurement. (**a**) Picture taken by the smartphone on the SBSM. (**b**); The grayscale spectrum of the rainbow picture; (**c**) Normalized absorption spectrum with and without sucrose solutions; (**d**) Relationship between wavelength shift and refractive index measured by the smartphone and the spectrometer.

A smartphone (MX5, MEIZU Inc, China) was used to capture the digital images. The smartphone has a powerful function in taking pictures. A master-mode was applied to capture images. The view of the master mode was presented in Figure S2. In this mode, some critical parameters (such as exposure time, photo-sensibility, focus, saturation) could be strictly controlled. The parameters were set up to avoid overexposure, which could lead to a maximum value for broadband (Figure S3). Five pictures were captured in each test to minimalize the influence of system error. A typical picture was presented in Figure 4a. The grayscale spectrum of this picture was presented in Figure 4b. The absorption spectrum of LSPR chip was calculated by Function (2):

(2) $$A=-\log\left(\frac{S-N}{R-N}\right),$$

where *S*, *N,* and *R* represented signal spectra, noise spectra, and reference spectra. 

In the absorption spectra of the LSPR chip, an absorption peak was presented. The absorption peak would shift when the refractive index around AuNPs changed. For example, sucrose solutions were injected into a micro-channel of an LSPR chip. The absorption spectrums before and after injecting were presented in Figure 4c. The absorption peak presented redshift, which means the refractive index around AuNPs increased from 1.0 in the air to 1.340 in 5% sucrose solutions. Different concentrations of sucrose solutions (0 to 40%) were tested by the SBSM. The refractive index of sucrose solutions was measured by the Abbe refractometer. The relationship between the wavelength shift and the refractive index was presented in Figure 4d. The image of nine major sensor points on the chip were captured by the SBSM to give out the red data points in Figure 4d. The blue data points were obtained via spectrometer test on the minor sensor points on the chip. Hence, the minor sensor points could be tested on spectrometer to prove the results of the major sensor points tested by the SBSM. The linear fitting result of the red data points in Figure 4d was as Function (3):

(3) S=161.0n−208.3,

where *S* and *n* represented wavelength shift and refractive index, respectively. The slope in the Function (3) means that the sensitivity of this LSPR chip was 161.0 nm/RIU.

### 2.6. Functionalization of AuNPs

**Figure 5:**
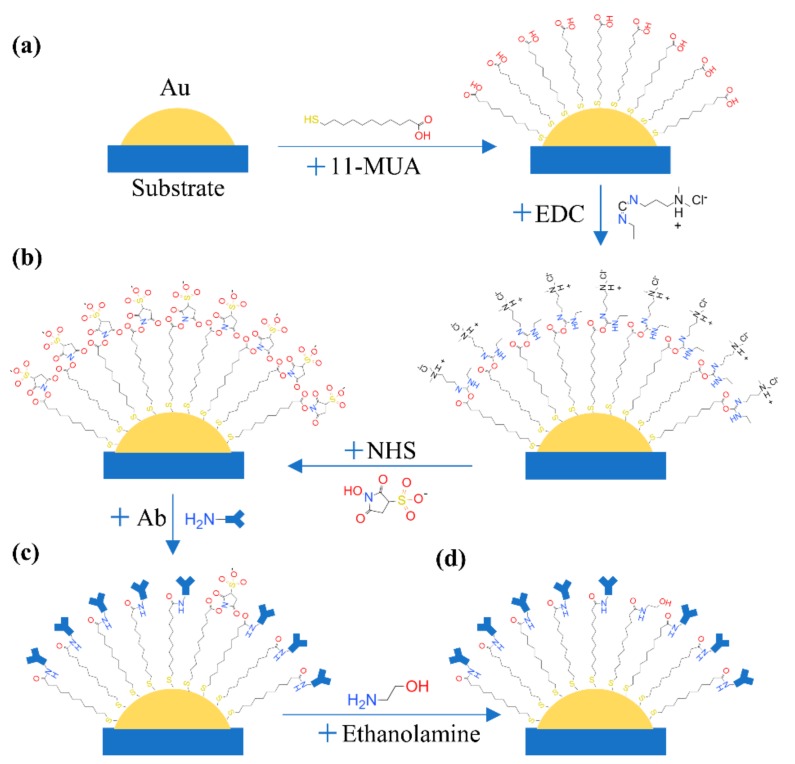
Functionalization of AuNPs. (**a**) The reaction between AuNPs and 11-MUA; (**b**) Active carboxyl via EDC and NHS; (**c**) Bonding antibody; (**d**) Quench amine reaction by ethanolamine.

AuNPs on chips should be functionalized to achieve the detection of protein. Firstly, AuNPs reacted with 11-MUA (10 mM, ethanol solutions) for 12 h to form an ordered self-assembled monolayer, which was presented in Figure 5a. The carboxyl of 11-MUA was active by EDC (75 mM, ethanol solutions) and NHS (25 mM, PBS) for 30 min. The volume ratio of EDC/NHS was 3. The processing of this reaction was presented in Figure 5b. The NHS ester could react with amines of protein to form a stable amide bond. Therefore, the specific antibody was injected into micro-channels to finish the functionalization (Figure 5c). The time of the antibody reaction was 2 h at room temperature. To avoid nonspecific binding, ethanolamine (1 M, PBS) was added to quench amine reaction of residue EDC or NHS for 2 h (Figure 5d). Before antigens reacting, the absorption spectrum of each sensor unit was measured to get the begin of the wavelength shift. Finally, the AuNPs were well functionalized and ready to detect cancer antigens. A well-functionalized chip can store at 4 °C for several weeks. Therefore, complex operations were finished by specialists in the laboratory. The customers only needed a one-step operation for the final test.

### 2.7. Detection of Cancer Biomarker in Clinical Serum Specimens

Two kinds of biomarkers related to breast cancer were chosen as target proteins. CA125 and CA15-3 have been reported to be related to breast cancer [29,30,31,32]. Channels on the LSPR chip were independent. Therefore, it is a customizable chip. Different kinds of antigens or different concentrations of antigen could be tested at the same time. Several concentrations of pure CA125 protein and CA15-3 protein were tested to set up a reference curve. Nine clinical serum specimens were tested to work out the concentration of CA125 and CA15-3 according to the reference curve. The serum was obtained from Qilu Hospital of Shandong University. The time of the antigen reaction was 1 h at 37 °C. Moreover, the usage of the sample is just about 10 μL. After the reaction, the absorption spectrum of each sensor unit was measured to find the end of the wavelength shift.

## 3. Results and Discussion

### 3.1. Reference Curve of CA125 and CA15-3 on the SBSM

**Figure 6:**
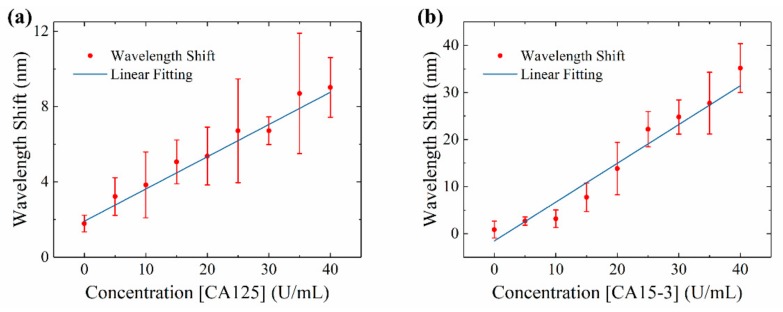
Reference curve of CA125 and CA15-3. (**a**) Reference curve of CA125 ranges from 0–40 U/mL; (**b**) Reference curve of CA15-3 ranges from 0–40 U/mL.

The reference curves of CA125 and CA15-3 were presented in Figure 6a,b. Due to the different structure and size between two proteins, the sensitivity of SBSM was different for CA125 and CA15-3. Based on the reference curve, the sensitivity was about 0.17 nm for per U/mL for CA125 and about 0.82 nm per U/mL for CA15-3. As the resolution of the SBSM was 0.71 nm, the LoD was 4.2 U/mL for CA125 and 0.87 U/mL for CA15-3. The LoD was not only limited by the measurement system and sensor chip but also related to the efficiency of biochemical reaction. A slight shift appeared when the concentration of protein was zero. Two causes could lead to this result: physical absorption and measuring error. During functionalization, some molecules were absorbed by AuNPs. Although buffers (PBS or ethanol) were used for flushing all channels between each step, there still were some molecules that were absorbed on AuNPs. These molecules could lead to a slight shift on the spectrum. However, measuring errors may also introduce unexpected shift. Therefore, all samples were tested several times to get the average results. There were nine major sensor units on one chip, which means one sample could be tested nine times at least. Additionally, there were eight minor sensor points along with each major sensor unit. These minor sensor points could be further tested on the spectrometer. Each chip had nine major sensor units and 72 minor sensor points; 81 tests could be operated on one chip.

### 3.2. Interference Testing

**Figure 7:**
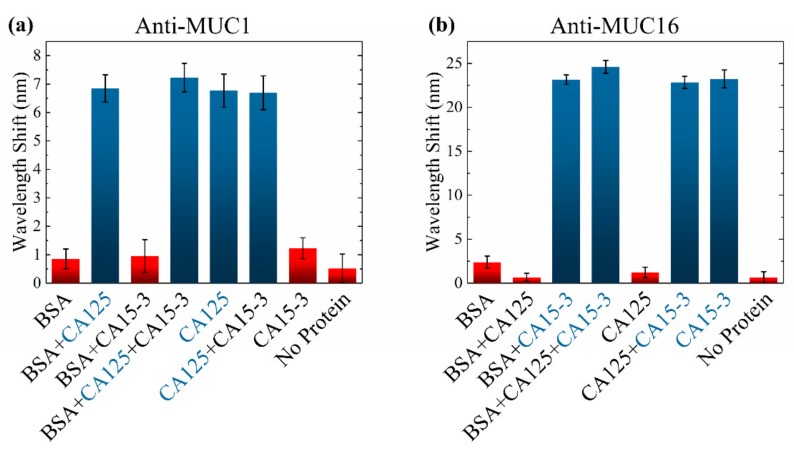
Results of interference testing when the LSPR chip was functionalized by (**a**) anti-MUC1 (for CA125); (**b**) anti-MUC16 (for CA15-3).

Clinical serum specimens contained a lot of kinds of proteins. Therefore, an interference testing was taken on the SBSM. Different kinds of pure protein were mixed and dissolved in PBS. To fully test the ability of specific detection, eight kinds of samples were prepared: BSA, BSA + CA125, BSA + CA125 + CA15-3, CA125, CA125 + CA15-3, CA15-3, and PBS without protein. The antibodies used in functionalization were anti-MUC1 (for CA125) and anti-MUC16 (for CA15-3). The concentration of BSA was 1%. The concentration of CA125 and CA15-3 was 30 U/mL. The results were presented in Figure 7. The concentration of BSA was much larger than the concentration of CA125 and CA15-3, but it did not influence the testing much. However, results of the non-protein samples indicated that the PBS may crystallize and adhere to the AuNPs. Therefore, sufficient flushing was significant during biological detection.

### 3.3. Results of Serum Measurement on the SBSM 

**Figure 8:**
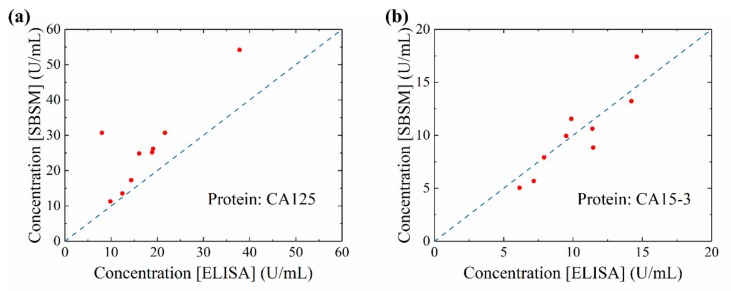
Data comparing results measured by the SBSM and results provided by the hospital. (**a**) The concentration of CA125 measured by the SBSM and ELISA (enzyme-linked immunosorbent assays); (**b**)The concentration of CA15-3 measured by the SBSM and ELISA. Blue dash line: reference line where concentrations measured by the SBSM and ELISA were equal.

Nine clinical serum specimens were measured by the SBSM. The measured concentration was compared with the concentration provided by the hospital, which was tested through ELISA, Figure 8a,b. The data point closed to the blue dash line means that two results were approximate. The concentration of CA125 measured by the SBSM was slightly higher than the concentration provided by the hospital. The cause of this result may be that the reference curve of CA125 was not exact. However, the total tendency of the result was correct. On the other hand, the concentration of CA15-3 measured by the SBSM fitted to the concentration provided by the hospital. Based on the result presented in Figure 8a,b, the SBSM could be regarded as reliable equipment to test protein concentration. The performance of the SBSM is enough for the POCT.

### 3.4. Relationship between Biomarkers and Cancer Early Stage

**Figure 9:**
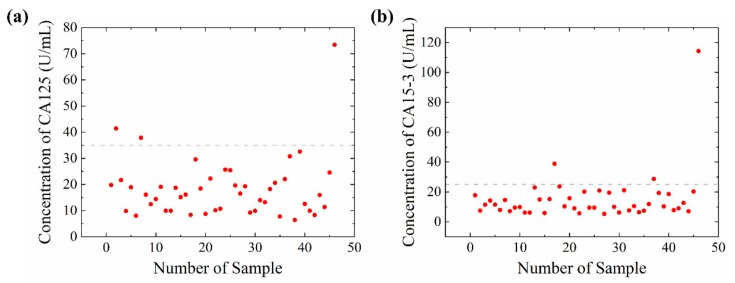
Concentration of CA125 and CA15-3 in serum samples. (**a**) The concentration of CA125. Gray dash line at 35 U/mL, which is the reference concentration of CA125 in clinic; (**b**) The concentration of CA15-3. Gray dash line at 25 U/mL, which is the reference concentration of CA125 in clinic.

A total of 46 patients’ serum samples were tested to research the relationship between the biomarker and cancer stage. The results were presented in Figure 9a,b. The gray dash line in Figure 9a,b were the reference concentration of CA125 and CA15-3 in clinic, respectively. The concentration of CA125 and CA15-3 in patients’ serum was not significantly higher than the reference. Only a few patients presented a relatively high concentration. However, in previous studies, long term monitor of the CA125 and CA15-3 in cancer patients’ serum presented a correlation between cancer stage and these biomarkers. Two reasons could explain the results. First, the concentration of CA125 and CA15-3 is relatively low in the serum of patients who are in the early stage of breast cancer. Second, the one time test could not give out an effective result. Long term monitoring should be carried out to diagnose the breast cancer stage of patients.

The SBSM could help to carry out the long-term monitor of patients. The usage of serum in the SBSM is low, which could reduce the pain of patients. The operation of the SBSM is easy, which could realize the POCT at home. Additionally, other biomarkers, such as CA27.29, could act as the biomarker to detect recurrence after primary breast cancer. The SBSM provides convenient equipment for the well-healed breast cancer patients to monitor the recurrence of breast cancer.

## 4. Conclusions

A multichannel spectrometer biosensor based on LSPR was firstly applied on smartphone platforms. Several well-designed attachments make a smartphone a cheap and convenient POCT equipment. The chips have nine sensor channels that could be customized for any biomarker. Each channel has one major sensor unit and eight minor sensor units. The major sensor unit is set for the SBSM, and minor sensor units are set for a further check on the spectrum. Moreover, the SBSM had modularized design. For different smartphone models, a specific smartphone stage could quickly assemble with other attachments. The performance of the SBSM was presented by testing different concentrations of sucrose solutions, protein solutions, and serum specimens. The SBSM could distinguish the different concentrations of sucrose solutions, and the sensitivity was 161.0 nm/RIU. The sensitivity could be improved by using a better LSPR chip. Uniform AuNPs fabricated by other methods (such as interference-lithography, anodic aluminum oxide template, electron beam lithography, and chemical synthesis) could effectively improve the sensitivity of LSPR chip. The resolution of the SBSM is 0.71 nm, which is comparable to the spectrometer in the laboratory. However, the resolutions of the SBSM could be improved by using a smaller micro-hole as well as a grating with more grooves. Light source with more power and a higher resolution camera could also help to improve the performance of the SBSM. The LoD was 4.2 U/mL for CA125 and 0.87 U/mL for CA15-3, which means the clinical reference concentration in the hospital could be distinguished. The results of the serum on SBSM were related to reports from the hospital. Long term monitoring was significant for primary breast cancer patients and well-healed patients. The advantage of the SBSM provided convenient equipment for patients. The SBSM was quick to use for non-professional people. The sample usage was only 10 μL, and the test time was only 1 h. Multi-testing of biomarkers could also reduce the test time for patients. Additionally, the attachments of the SBSM were portable. Except for the smartphone, the total volume of the attachment was less than 70 cm^2^, which is suitable for a portable POCT. With the help of other hybrid manufacturing processes, the cost of the SBSM could be reduced, and the rate of production could be improved. For example, the substrate and microfluidic chip could be fabricated via polymer injection molding, which is more suitable for quantity production. The grating could be fabricated via nanoimprint lithography, which could reduce the cost of the SBSM. The lens could be fabricated via PDMS molding, and other attachments could be fabricated via three-dimensional (3D) printing. Thus, the SBSM could be a promising POCT equipment in the future. 

## Supplementary Materials

The following are available online at https://www.mdpi.com/1424-8220/20/2/446/s1. Table S1: Information regarding materials, chemicals, biologicals, and components; Figure S1: Workflow of the Python program during image analyzation; Table S2: The spectral calibration results of all channels; Figure S2: View of master mode on the smartphone; Figure S3: An example of overexposure.
