# Supplementary material for: Smartphone Biosensor System with Multi-Testing Unit Based on Localized Surface Plasmon Resonance Integrated with Microfluidics Chip
Source: Sensors (Basel). 2020 Jan 13;20(2):446. doi: 10.3390/s20020446 (PMC7014366; doi:10.3390/s20020446)
Supplement: Supplementary file 1 [file sensors-20-00446-s001.pdf]

Supporting Information: Smartphone Biosensor System with Multi-Testing Unit Based on Localized Surface Plasmon Resonance Integrated with Microfluidics Chip

Zhiyuan Fan [1,2], Zhaoxin Geng [1,3,*], Weihao Fang [1,2], Xiaoqing Lv [1], Yue Su [1,2], Shicai Wang [4] and Hongda Chen [1,2]

1 State Key Laboratory of Integrated Optoelectronics, Institute of Semiconductors, Chinese Academy of Sciences, Beijing 100083, China; Fanzhiyuan@semi.ac.cn (Z.F.); fwh@semi.ac.cn (W.F.); lvxiaoqing284@semi.ac.cn (X.L.); suyue@semi.ac.cn (Y.S.); hdchen@semi.ac.cn (H.C.)
2 Center of Materials Science and Optoelectronics Engineering, University of Chinese Academy of Sciences, Beijing 100049, China
3 School of Information Engineering, Minzu University of China, Beijing 100081, China
4 State Key Laboratory of Crystal Materials, Shandong University, Jinan 250100, China; cjchao@semi.ac.cn
* Correspondence: Zxgeng@muc.edu.cn

1. The Workflow of the Python Program

The software used to write and run the Python program is PyCharm (version 2019.1.3). The version of Python is 3.7.3. The packs and functions used in the Python program including opencv (version 3.4.2), numpy (version 1.16.4), matplotlib (version 3.1.0), and scipy (version 1.3.0).

Origin images were captured by the smartphone during the test. To avoid system errors, at least five images were captured each time. It is a repetitive and hard work to read out and analyze a huge number of images. Therefore, a python program was written to finish the work of analyzation. For each image, the workflow of the Python program was presented as follows.

Border region of the image is totally black, which is useless for sensor. However, the black border region would increase the calculation time. Therefore, each image is cropped to define the sensor region as shown in Figure S1a.

The colorful image in Figure S1a was transferred into the grayscale image, Figure S1b. The calculation function was as follows:

$$Gray = 0.299R + 0.587G + 0.114B, \quad (1)$$

where *Gray* represents the grayscale of one pixel. *R*, *G*, and *B* represent the red, green, and blue scale (0–255) of the pixel. The *R*, *G*, *B* were recorded by the CMOS (Complementary Metal-Oxide-Semiconductor Transistor) sensor.

The images could be regarded as a matrix of pixel. The *x* axis was defined as the direction perpendicular to the rainbow bar, and the *y* axis was defined as the direction parallel to the rainbow bar. The sum of each column was presented in Figure S1c. Each peak in Figure S1c presented the center of each rainbow bar respectively.

The sensor regions were redefined based on the center of each rainbow bar. The new sensor regions were nine rectangles that contained the center of each channel. In Figure S1d, the new sensor regions were labeled by the white line.

For each sensor region, the sum of each row related to the intensity of different wavelengths. Figure S1e presented a typical intensity-pixel position curve.

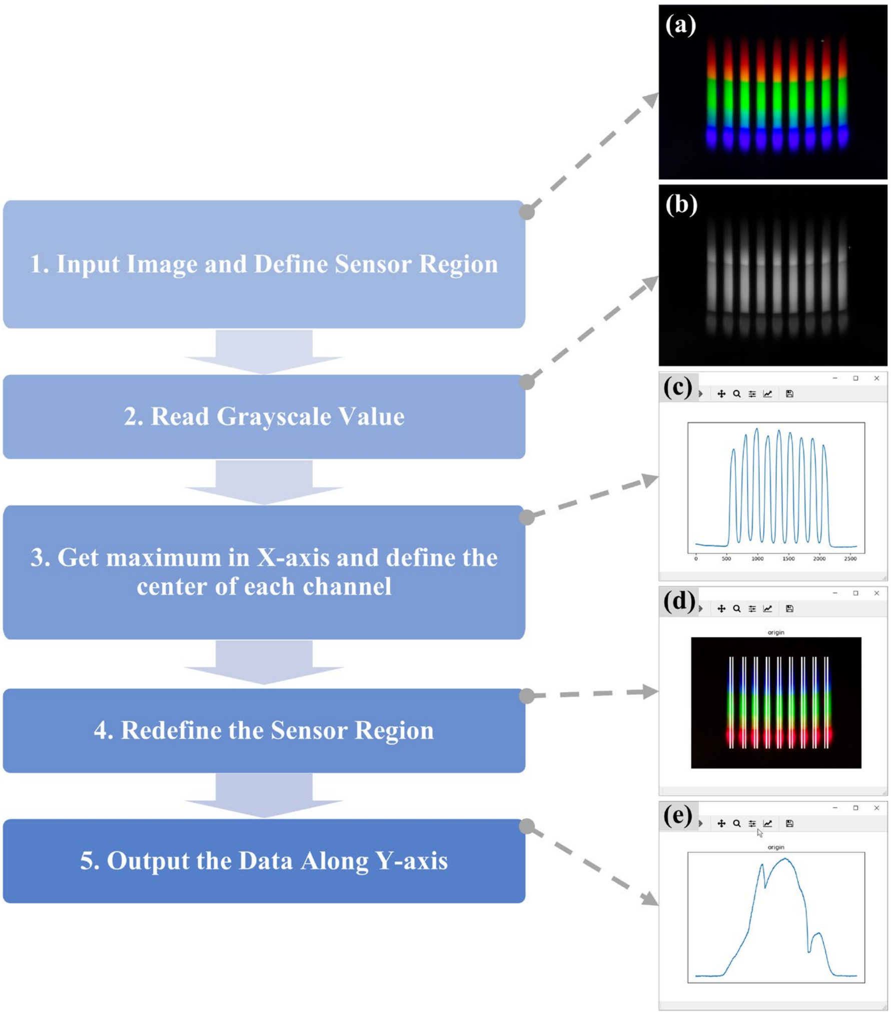

Figure S1. Workflow of the Python program during image analyzation. (**a**) Get rid of the black border region and define the sensor region; (**b**) calculate the grayscale of the image; (**c**) get maximum in x axis and define the center of each channel; (**d**) redefine the sensor region; (**e**) output the spectrum of the image.

2. Materials, Chemicals, Biologicals, and Components

Table S1. The information of materials, chemicals, biologicals, and components.

Category		Name	Type	Specification	Provider or Producer
Materials	1	Quartz substrate	JGS1	20 × 20 × 0.5 mm	Wuxi Crystal and Optical Instrument Co. Ltd., China
	2	Polydimethylsiloxane (PDMS)	SYLGARD™ 184	Two-part mixing, 10:1, heat cure	Dow Corning, USA.
Chemicals	3	Sulfuric acid	10021618	AR, 95.0%~98.0%	Sinopharm Chemical Reagent Beijing, Co. Ltd., China
	4	Hydrogen peroxide	10011218	AR, ≥30.0%	
	5	Sucrose	10021463	AR	
	6	Deionized (DI) water	F0020	Endotoxin ≤0.005 EU/ml	Solarbio Beijing, Co. Ltd., China
	7	Ethanol	E111993	HPLC, ≥99.8%	Shanghai Aladdin Bio-Chem Technology Co., LTD., China
	8	Ethanolamine	398136	ACS reagent, ≥99.0%	Sigma–Aldrich, USA
	9	Thiolated alkane 10-carboxy-1-decanethiol (11-MUA)	674427	98%	
	10	1-ethyl-3-(3-dimethylamino-propyl) carbodiimide-HCl (EDC)	E6383	Crystalline	
	11	N-hydroxy succinimide (NHS)	56480	Purum, ≥97.0% (T)	
	12	Bovine serum albumin (BSA)	SW3015	5%	
	13	Phosphate buffer solution (PBS, 1X)	SH30256.01	0.0067 M (PO_4), pH: 7.4	Thermo Fisher, USA
Biologicals	14	Cancer antigen 125 protein (CA125)	MUC16-1H	Purity: >95% (SDS-PAGE)	Creative BioMart, USA.
	15	Cancer antigen 15-3 protein (CA15-3)	MUC1-376H	Purity: Control Grade	
	16	Anti-MUC16 antibody	ab134093	Purity: Tissue culture supernatant	Abcam, USA
	17	Anti-MUC1 antibody	ab109185	Purity: Protein A purified	
Components	18	Lens 1	35-697	1.0 mm Dia., 2.0 mm FL, VIS-EXT Coated	Edmund Optics Inc., USA
	19	Lens 2	88-710	20.0 mm Dia., 25.0 mm FL, VIS-EXT,	
	20	Transmission grating	49-582	1200 Grooves, 25 mm Sq, 36.9° Blaze Angle	
	21	LED	Customized	3V, 15 mA, White light	Hongrui Backscatter light, China
	22	Micro-hole array	Customized	500 μm Dia., Film Photomask	MICROCAD PHOTO-MASK LTD., China
	23	Smartphone	MX5	20.7 million pixels	MEIZU Inc, China

3. The Calibration Result of Each Channel

Due to the difference of the physical structure and the optical deviation, the calibration results at different positions were different. Table S2 listed the spectral calibration results of all channels. The intercept of the linear-regression results ranged from 353.6037 to 356.9689, and the slope of the linear-regression results ranged from 0.18897 to 0.19322. The difference of intercept and slope between channels could not be ignored. Therefore, different intercepts and slopes were applied to calculate the spectrum data for different channels. However, the adjusted coefficient of determination (Adj. R-Square) approached 1 at the spectral calibration results of all channels, which means that the system was highly precise at anywhere on the sensor region.

Table S2. The spectral calibration results of all channels.

Channels	Intercept		Slope		Statistics
	Value	Standard Error	Value	Standard Error	Adj. R-Square
1	355.8212	0.12033	0.18973	1.18E-04	1
2	356.8013	0.10345	0.19113	1.02E-04	1
3	356.9593	0.19709	0.19241	1.96E-04	1
4	356.9689	0.24857	0.19321	2.49E-04	1
5	356.8323	0.4333	0.19322	4.33E-04	0.99999
6	356.0742	0.35023	0.19306	3.49E-04	0.99999
7	355.6584	0.05379	0.19192	5.31E-05	1
8	355.1502	0.05421	0.1902	5.29E-05	1
9	353.6037	0.01179	0.18897	1.14E-05	1

4. The camera Control Software in Smartphones

To get uniform and reliable data, the camera should be strictly controlled to take photos. The main factors in taking photos include exposure time, photosensitivity (ISO) and focal length. Most smartphones have software to control their cameras. The smartphone used in this study has a built-in function to control the camera named master mode. The mater mode could adjust exposure time, ISO, and focal length accurately. In Figure S2, the uses of the master mode were presented. The slide bar on the interface can be dragged to adjust the numerical value of the factors.

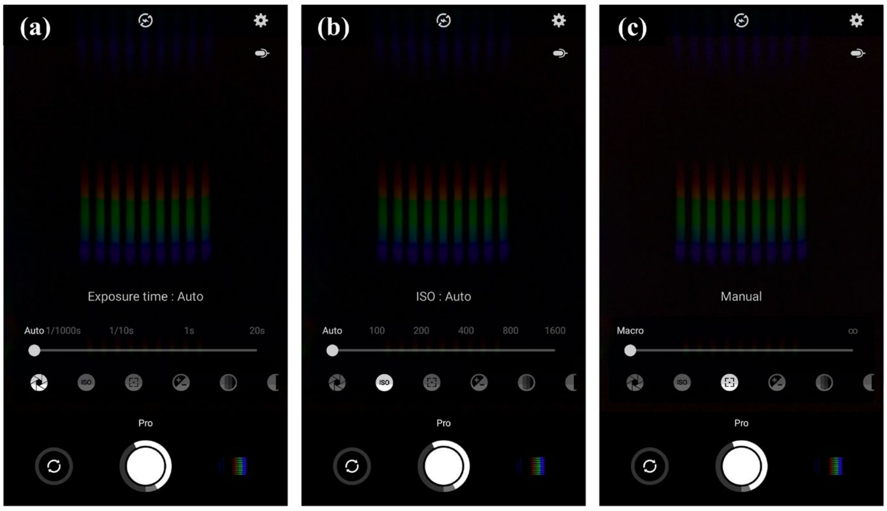

Figure S2. The view of master mode on the smartphone. (**a**) Adjust the exposure time; (**b**) adjust the photosensitivity (ISO); (**c**) adjust the focal length.

5. Overexposure

The intensity recorded by the sensor could be saturated if long exposure time was applied. However, higher ISO may also produce an overexposed image. In Figure S3, an example of overexposure was presented. The white dash line in Figure S3a indicated the center of the fifth channel, whose intensity data of RGB and grayscale were presented in Figure S3b,c. In Figure S3b, the spectrum of the green and blue channels showed flat tops where the sensor was saturated, and the grayscale in Figure S3c also showed a flat top. These flat tops would lead to wrong results. Therefore, the exposure time and the ISO should not be too high. The exposure time and the ISO should be adjusted according to the size of micro-hole and power of light source before testing. During a series of tests, the exposure time and the ISO should be same.

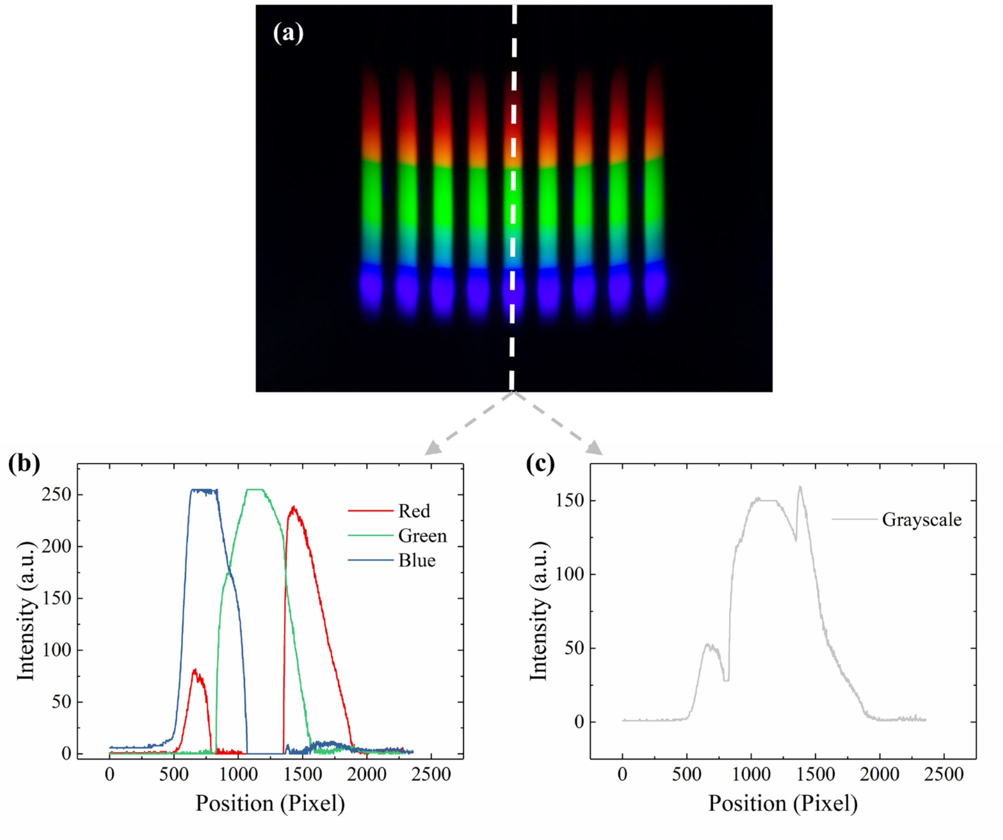

Figure S3. An example of overexposure. (**a**) Overexposed picture; (**b**) spectrum of three channels of color (red, green, and blue); (**c**) spectrum of grayscale.
